# Effect of Low Concentration of Nitroxides on SH-SY5Y Cells Transfected with the Tau Protein

**DOI:** 10.3390/ijms242316675

**Published:** 2023-11-23

**Authors:** Grzegorz Bartosz, Natalia Pieńkowska, Kacper Kut, Bogumił Cieniek, Ireneusz Stefaniuk, Izabela Sadowska-Bartosz

**Affiliations:** 1 Laboratory of Analytical Biochemistry, Institute of Food Technology and Nutrition, College of Natural Sciences, Rzeszow University, 4 Zelwerowicza Street, 35-601 Rzeszow, Poland; gbartosz@ur.edu.pl (G.B.); natalia.pien@gmail.com (N.P.); kacper.kut@onet.pl (K.K.); 2 Institute of Materials Engineering, College of Natural Sciences, University of Rzeszów, 35-310 Rzeszów, Poland; bcieniek@ur.edu.pl (B.C.); istefaniuk@ur.edu.pl (I.S.)

**Keywords:** nitroxides, TEMPO, TEMPOL, TEMPAMINE, ABTS decolorization, DPPH, FRAC, CUPRAC, SH-SY5Y cells, glutathione

## Abstract

Nitroxides, stable synthetic free radicals, are promising antioxidants, showing many beneficial effects both at the cellular level and in animal studies. However, the cells are usually treated with high millimolar concentrations of nitroxides which are not relevant to the concentrations that could be attained in vivo. This paper aimed to examine the effects of low (≤10 μM) concentrations of three nitroxides, 2,2,6,6-tetramethylpiperidin-1-oxyl (TEMPO), 4-hydroxy-TEMPO (TEMPOL) and 4-amino-TEMPO (TEMPAMINE), in pure chemical systems and on SH-SY5Y cells transfected with the human tau protein (TAU cells), a model of chronic cellular oxidative stress, and transfected with the empty plasmid (EP cells). All nitroxides were active in antioxidant-activity tests except for the 2,2′-azinobis-(3-ethylbenzthiazolin-6-sulfonate) radical (ABTS^•^) decolorization assay and reduced Fe^3+^, inhibited autoxidation of adrenalin and pyrogallol and oxidation of dihydrorhodamine123 by 3-morpholino-sydnonimine SIN-1. TEMPO protected against fluorescein bleaching from hypochlorite, but TEMPAMINE enhanced the bleaching. Nitroxides showed no cytotoxicity and were reduced by the cells to non-paramagnetic derivatives. They decreased the level of reactive oxygen species, depleted glutathione, and increased mitochondrial-membrane potential in both types of cells, and increased lipid peroxidation in TAU cells. These results demonstrate that even at low micromolar concentrations nitroxides can affect the cellular redox equilibrium and other biochemical parameters.

## 1. Introduction

Nitroxides are synthetic antioxidants. They are stable organic radicals, with the nitroxyl group >N–O^●^ containing an unpaired electron. Their stability is secured with bulky (usually methyl) groups surrounding the nitroxyl group. By undergoing one-electron-transfer reactions, nitroxides can act as hydrogen-donor antioxidants, directly scavenge free radicals, and oxidize transition metal ions, thus preventing the Fenton reaction and peroxide decomposition by metal ions [1]. In these one-electron-transfer reactions, nitroxides are reduced to the corresponding hydroxylamines via one-electron-transfer reactions or enzyme-related mechanisms [2,3]. The hydroxylamines can function as typical reducing agents, like vitamin C or E, to scavenge oxidants [1] and to be oxidized back to nitroxides [4,5,6,7]. In cells treated with TEMPO, the nitroxide concentration (assessed using the EPR signal intensity) decreases over time because of cell-mediated reduction, mainly to the corresponding hydroxylamine [3,8,9].

Nitroxides are also reversibly oxidized to oxoammonium cations [4,5,10,11], and the nitroxide/oxoammonium couple can efficiently catalyze the decomposition of superoxide [11,12,13] showing a pseudo-enzymatic superoxide dismutase (SOD) activity, and inducing catalase-like activity in heme proteins [7,14]. Consequently, all three oxidation states may be present and active in complex biological systems [6,14,15,16]. Both nitroxides and hydroxylamines can protect cells exposed to superoxide, H_2_O_2_, or organic peroxides [3]. Seemingly, the nitroxide is the primary protective species, though lesser contributions from the hydroxylamine cannot be dismissed.

2,2,6,6-Tetramethylpiperidine-1-oxyl (TEMPO), one of the most commonly used nitroxides, has a variety of uses related to its role as a radical scavenger and stabilizer. TEMPO is also broadly employed in organic synthesis for the oxidation of primary and secondary alcohols to the corresponding aldehydes and ketones. TEMPO catalytic systems (e.g., ruthenium/TEMPO and copper/TEMPO) were reported to enable efficient oxidation of a broad range of primary alcohols, which facilitated their widespread use in synthetic chemistry [17,18].

A range of protective effects of nitroxides on cells, especially under conditions of oxidative stress, has been described. 4-Hydroxy-TEMPO (TEMPOL; 0.1 and 0.2 mM) protected HepG2 cells against arachidonic acid toxicity [19]. (TEMPOL) (1–2 mM) was reported to inhibit megamitochondria formation and apoptotic changes in rat-liver RL-34 cells induced by chloramphenicol [20]. 4-O-acetyl-TEMPO and TEMPOL (7 mM) protected *Vibrio harveyi* bacteria against oxidative-stress mediated growth inhibition inferred by cumene hydroperoxide, *tert*-butyl hydroperoxide and ferrous sulfate but not hydrogen peroxide, although they slightly inhibited the growth of control cells [21]. TEMPOL (5–100 mM) protected Chinese hamster cells against ionizing radiation under aerobic but not under anaerobic conditions [22]. TEMPOL (3 mM) protected HT22 cells against buthionine sulfoximine (BSO)-induced decrease of total antioxidant activity and SOD activity, and the levels of SOD1 and SOD2, oxygen consumption, ATP level, and mitochondrial potential [23]. 4-Amino-TEMPO (TEMPAMINE) was effective in protecting human fibroblasts against the loss of viability induced by hydrogen peroxide [24]. TEMPOL was protective against the cytotoxic and mutagenic effects induced by hydrogen peroxide and hypoxanthine/xanthine oxidase in mouse lymphoma cells [25]. TEMPO (50–1000 μM) protected J774.A1 murine macrophage cells against Rose Bengal—photosensitized viability loss and dityrosine and DOPA formation. However, significant protective effects were observed only for concentrations > 100 μM (protection of cell viability and inhibition of dityrosine formation) and >250 μM (protection against DOPA formation) [26]. TEMPO (3 mM) displayed a genoprotective effect in the yeast *Saccharomyces cerevisiae* by reducing the number of DNA double-strand breaks in cells [27].

However, since nitroxides also can be converted into highly oxidizing oxoammonium, they can have pro-oxidant activity under certain conditions and induce adverse effects in cells [28]. Toxicity of nitroxide free radicals, has been reported in studies conducted with both bacteria and mammalian cells. For example, nitroxides were mutagenic in *Salmonella typhimurium* strains TA104 and TA100 [29,30]. Some nitroxides manifest pro-oxidant effects by increasing the cellular hydrogen peroxide concentration [31,32]. Treatment of TK6 human lymphoblastoid cells with 0.9–2.3 mM TEMPO increased the frequency of both micronuclei (a marker for clastogenicity) and hypodiploid nuclei (a marker of aneugenicity) in a dose-dependent manner. Within this dose range, TEMPO induced reactive oxygen species and decreased glutathione levels [33]. TEMPOL inhibited cell growth in a panel of human and rodent cultured cell lines, possibly by triggering an apoptotic mechanism with a consistent preference for neoplastic cells, with IC_50_ (the half maximal inhibitory concentration) values of 0.2–1.1 mM depending on the cell type [34]. TEMPO, TEMPOL, and TEMPAMINE (0.2–2 mM) decreased the ascorbate content of erythrocytes and lactate dehydrogenase activity and increased the oxidation of hemoglobin to methemoglobin [35]. TEMPO inhibited the growth of the yeast *S. cerevisiae*. The growth of the wild-type BY4741 strain was inhibited by 3 and 5 mM TEMPO; SOD-deficient strains were more sensitive, with their growth being inhibited even by 100 μM TEMPO [27].

The presence of different substituent groups may result in modified biological effects. The substituents affect redox properties of the nitroxides, the sequence of redox potentials being TEMPO < TEMPOL < TEMPAMINE [13]. TEMPO, TEMPOL, 4-oxo-TEMPO, and TEMPAMINE showed different toxicity in human HaCaT keratinocytes [36]. Following a 24 h treatment, TEMPO was the most cytotoxic nitroxide, with an IC_50_ value of 2.66 mM, while TEMPOL showed the least cytotoxicity with an IC_50_ value of 11.4 mM [25].

In these experiments, high (millimolar) nitroxide concentrations were usually used. However, if in vivo applications of nitroxides are to be considered, lower nitroxide concentrations, available in vivo, deserve closer attention. Although the plasma nitroxide concentrations achieved in animal studies, in which TEMPOL was administered via drinking water or in food at 10 mg/g chow, were shown to increase life span and decrease tumor incidence were 90–100 μM [37], nitroxides are rapidly metabolized and excreted. Moreover, these nitroxide doses applied were extremely high, and the application of lower doses can be expected in future experiments.

Other antioxidants were found to act effectively in cellular experiments in lower concentrations. A study of the effects of rosmarinic acid, ampelopsin, and amorfrutin A on the proliferation of fibroblasts showed optimal effects of these compounds at concentrations ≤ 1 μM [38]. Methylene Blue, 1 and 10 μM, protected primary rat retinal ganglion cells against hypoxia, rotenone, and staurosporine [39]. This compound, at a concentration of 100 nM, improved the proliferation of fibroblasts and reduced symptoms of cellular aging [40], and at a concentration of 200 nM was found to improve the expansion and differentiation potential of mesenchymal stem cells [41].

It is, therefore, of interest to check whether low concentrations of nitroxides, attainable in in vivo experiments for a prolonged time (≤10 μM), can affect chosen reactions in chemical systems and biochemical parameters of cells. We compared two SH-SY5Y cell lines, one expressing the human tau40 protein and showing symptoms of permanent oxidative stress [42], and a control line, both transfected with an empty plasmid.

## 2. Results

### 2.1. Antioxidant Properties of Nitroxides

From the results of the antioxidant assays, the antioxidant activity (expressed in moles of Trolox Equivalents (TE) per mol of nitroxide) and the number of moles of indicator radicals or Fe^3+^ ions reduced by one mole of a nitroxide were calculated (Table 1).

In the azinobis-(3-ethylbenzthiazolin-6-sulfonate radical) (ABTS^•^) decolorization assay, no increase in the extent of ABTS^•^ reduction between 1 min and 30 min was observed (not shown), which suggests that the low values of antioxidant activity are not due to the slow reaction with ABTS^•^ but that, most probably, the nitroxides do not reduce ABTS^•^, and the small extent of reduction measured is due to impurities (perhaps hydroxylamines) present in the commercial nitroxide preparations. The nitroxides were reactive in the DPPH^•^ decolorization, FRAP, and CUPRAC assays: one mole of a nitroxide reducing 0.88–1.20 mole of DPPH^•^ and 0.64–1.06 mole of Fe^3+^ under the conditions of the assay. It was not possible to estimate reliably the number of nitroxide radicals reacting per one Cu^2+^ ion as the absorption coefficient of the Cu(I) neocuproine complex could not be independently determined, but the antioxidant activities of nitroxides were not much lower than that of ascorbate, suggesting a reactivity of at least one mole of a nitroxide per mole of Cu^2+^ ions.

Interestingly, the nitroxides were able to oxidize Fe^2+^ ions at pH 7 in a “reversed FRAP assay” (Table 2), though no Fe^2+^ oxidation was observed under conditions of standard FRAP assay (acetate buffer, pH 3.6, the presence of TPTZ; not shown). The differences in the oxidation rates between the nitroxides have different directions for the two concentrations used, so, although statistically significant, they do not seem to have real chemical significance.

### 2.2. Effect of Nitroxides on the Adrenalin and Pyrogallol Autoxidation

**Figure 1:**
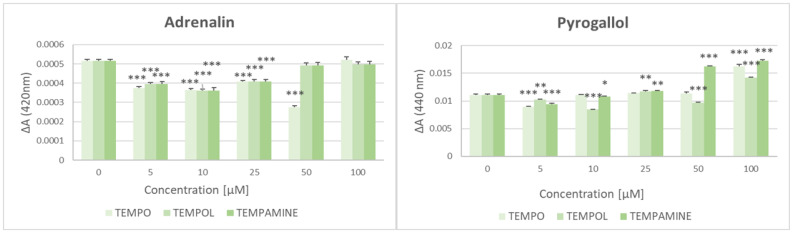
Effect of TEMPO, TEMPOL, and TEMPAMINE on the autoxidation of adrenalin and pyrogallol. * *p* < 0.05, ** *p* < 0.01, *** *p* < 0.001 (with respect to samples containing no nitroxide).

All the nitroxides studied at the lower concentrations used decreased the rate of autoxidation of pyrogallol and adrenalin, but this effect disappeared at higher nitroxide concentrations (5 and 10 μM) which even increased the rate of autoxidation of pyrogallol (Figure 1).

### 2.3. Effect of Nitroxides on the Oxidation of Dihydrorhodamine 123

**Figure 2:**
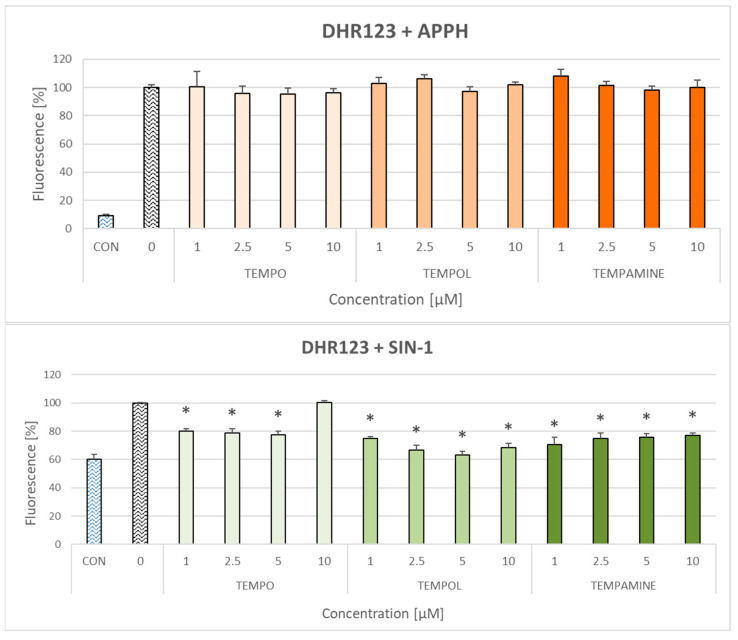
Effect of TEMPO, TEMPOL, and TEMPAMINE on the oxidation of DHR123 by AAPH and SIN-1. The fluorescence of the oxidation product in the absence of nitroxides assumed as 100%. CON, fluorescence in the absence of the oxidizing agent. * *p* < 0.05 (with respect to samples treated with an oxidant but containing no nitroxide).

Nitroxides did not affect the oxidation of dihydrorhodamine 123 (DHR123) by AAPH—a generator of peroxyl radicals—but inhibited the oxidation of DHR123 caused by 3-morpholino-sydnonimine (SIN-1)—a generator of peroxynitrite (Figure 2).

### 2.4. Effect of Nitroxides on the Bleaching of Fluorescein with Hypochlorite

**Figure 3:**
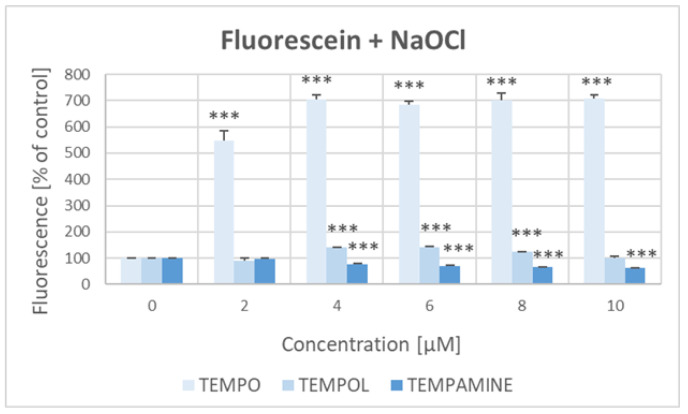
Effect of nitroxides on the bleaching of fluorescein with hypochlorite. The fluorescence of fluorescein treated with NaOCl in the absence of nitroxides assumed as 100%. *** *p* < 0.001 (with respect to samples treated in the absence of a nitroxide).

The bleaching of fluorescein with NaOCl was considerably inhibited by TEMPO, and, to a lesser extent, 4–8 μM TEMPOL, but was enhanced by 4–10 μM TEMPAMINE (Figure 3).

### 2.5. Toxicity of Nitroxides to SH-SY5Y Cells

**Figure 4:**
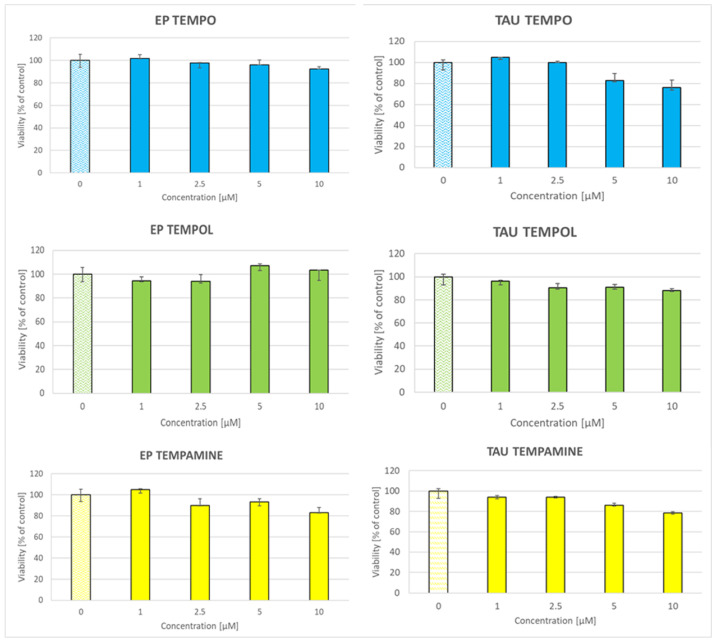
Effect of nitroxides on the survival of SH-SY5Y cells transformed with empty plasmid (EP) and plasmid coding for the human TAU40 protein (TAU).

At the low concentrations employed, nitroxides did not exert significant toxicity toward the SH-SY5Y cells (Figure 4).

### 2.6. Reduction of Nitroxides by SH-SY5Y Cells

**Figure 5:**
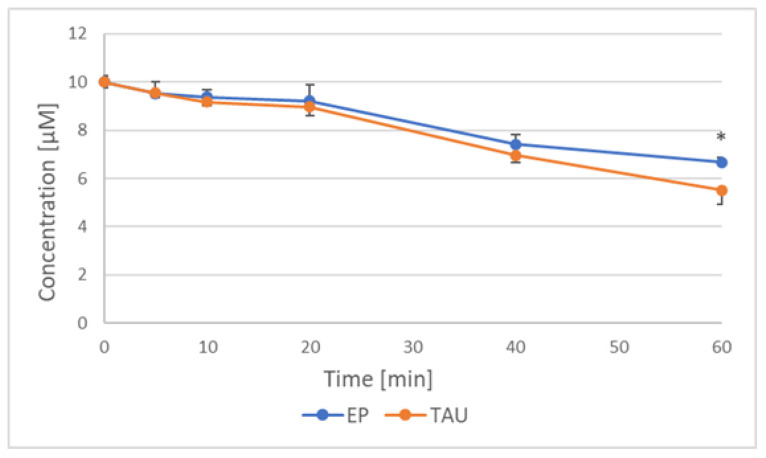
Loss of TEMPO EPR signal during incubation with EP and TAU cells with 10 μM nitroxide. * *p* < 0.05 (EP vs. TAU cells).

A time-dependent loss of ESR signal of nitroxides was observed during incubation of nitroxides with SH-SY5Y cells transfected with the human Tau protein (TAU cells) and cells transfected with the empty plasmid (EP cells), due to nitroxide reduction to non-paramagnetic derivatives (Figure 5).

The rate of loss of EPR signal of nitroxides during incubation with SH-SY5Y cells is compared in Table 3. The rate of reduction of various nitroxides did not differ significantly but the rate of reduction was higher for TAU than for EP cells.

### 2.7. Effect of Nitroxides on the Level of Reactive Oxygen Species

**Figure 6:**
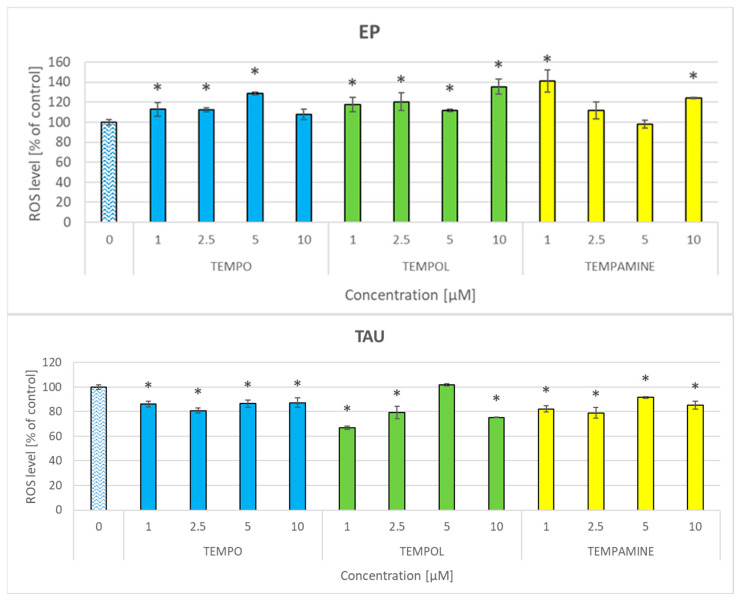
Effect of nitroxides on the level of ROS, estimated with dihydroethidium, in EP and TAU cells. * *p* < 0.05.

The nitroxides affected the level of reactive oxygen species (ROS) in the cells, though the effects were small and not exceeding (with one exception) 20%. Generally, all the nitroxides elevated the ROS level in EP cells or had no effect, while in TAU cells, characterized by a higher ROS level, and the nitroxides decreased the ROS level or had no effect. There was no concentration–effect dependence in the concentration range applied (Figure 6).

### 2.8. Effect of Nitroxides on the Glutathione Content of the Cells

**Figure 7:**
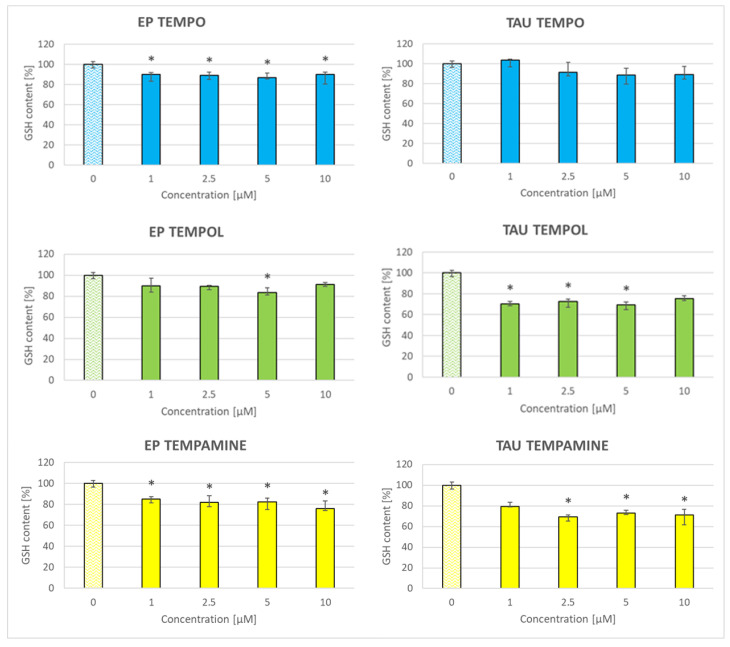
Effect of nitroxides on the glutathione content of EP and TAU cells. * *p* < 0.05.

The nitroxides decreased the glutathione content in SH-SY5Y cells, except for TEMPO in the case of TAU cells (Figure 7). The magnitude of this loss reached about 10% in EP cells treated with TEMPO, about 20% in EP cells treated with TEMPOL and TEMPAMINE and 30% in TAU cells treated with TEMPOL and TEMPAMINE.

### 2.9. Effect of Nitroxides on Lipid Peroxidation

**Figure 8:**
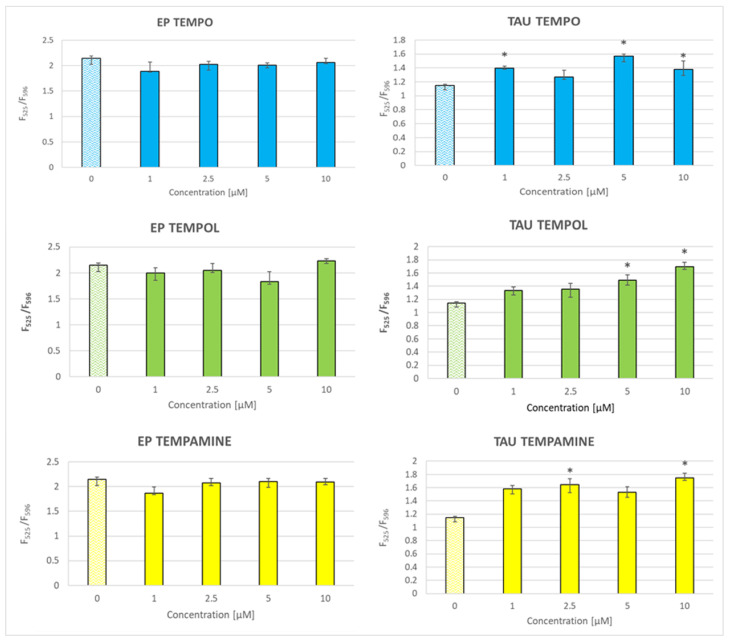
Effect of nitroxides on the level of lipid peroxidation in EP and TAU cells. * *p* < 0.05.

The level of lipid peroxidation, estimated with BODIPY C11, was enhanced by nitroxides in TAU but not EP cells (Figure 8).

### 2.10. Effect of Nitroxides on the Mitochondrial-Membrane Potential

**Figure 9:**
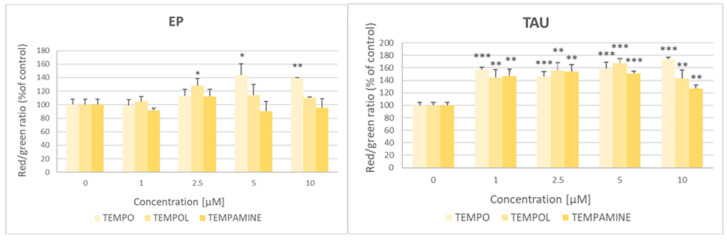
Effect of nitroxides on the mitochondrial potential of EP and TAU cells. C. * *p* < 0.05, ** *p* 0.01, *** *p* < 0.001.

Nitroxides enhanced the red-to-green fluorescence ratio of the JC-1 probe responsive to the mitochondrial potential, indicative of an increase in the mitochondrial potential, especially in the TAU cells where this effect was observed for the entire concentration range applied (1–10 μM); in the case of EP cells, this effect was seen only for 2.5 μM TEMPOL and 5 and 10 μM TEMPO (Figure 9).

## 3. Discussion

The presented results demonstrate that even at low (1–10 μM) concentrations nitroxides can exert significant effects in both pure chemical and cellular systems, exhibiting both antioxidant and prooxidant effects.

As nitroxides show antioxidant properties, it was of interest to estimate their behavior in standard antioxidant assays. Nitroxides were essentially unreactive in the ABTS^•^ decolorization assay but reacted in the other assays applied. This behavior of nitroxides is not easy to explain, since the standard redox potential of the of the ABTS^•^/ABTS redox couple was reported to be 0.68 V [43], while that of the (Cu(II)(neocuproine)/Cu(I)neocuproine) to be 0.60 V [44]. Thus, the reduction of ABTS^•^ should be thermodynamically favored over the Cu(II)(neocuproine) reduction, though these data are hard to reconcile with the reported standard redox potential of the TEMPO^+^/TEMPO redox couple of 0.71 V [45]. It seems that the values of the redox potentials of these systems require careful re-examination. The lack of nitroxide reactivity with ABTS may be due to steric hindrance rather than a thermodynamic restriction.

In a recent study, the reactivity of TEMPO comparable to those of ascorbic acid and glutathione in the ABTS^•^ decolorization assay was reported [27]. However, the reliability of those data is compromised by the unusually low reactivities of both ascorbate and glutathione in this assay (0.056 and 0.049 mol TE/mol, respectively, much lower than the values commonly found).

The reactivity of nitroxides in the FRAP and CUPRAC assays is somewhat surprising, since nitroxides are thought to oxidize transition metal ions, and in this way exert an antioxidant effect by preventing the Fenton reaction [1]. However, we found that the nitroxides were able to oxidize Fe^2+^ in a “reversed FRAP assay” at pH 7. The standard redox potential of the Fe^3+^/Fe^2+^ couple was found to be 0.77 V at pH 2.0. However, the redox potential of the Fe^3+^/Fe^2+^ couple is strongly dependent on the ligand and, for example, is 0.375 V for ferric/ferrous citrate at near-neutral pH [46]. Thus, the direction of the reaction between nitroxides and Fe ions may be determined by the conditions, first of all, by the character of ligands. Similarly, the standard redox potential of the Cu^2+^/Cu^+^ couple is 0.17 V, much lower than that of the copper–neocuproine complex [44]. Therefore, the reactivity of nitroxides in the metal-ion-based antioxidant assays does not preclude oxidation of the transition metal ions under different conditions, depending on the nature of the ligands.

Low concentrations of nitroxides can be expected to interfere with redox reactions in which either the concentrations of reagents or the steady-state concentrations of critical reaction intermediate(s) are low. In this study, the effect of nitroxides on the autoxidation of adrenalin and pyrogallol was examined. Both reactions are dependent on the intermediacy of superoxide radical anion [47,48], so nitroxides could be expected to interfere due to their pseudoenzymatic activity of superoxide dismutase [49,50]. Lower concentrations of nitroxides applied inhibited autoxidation of both compounds, but this effect disappeared at higher concentrations of nitroxides. Both reactions are rather complex and, apart from superoxide, fortuitous metal ions contribute to the course of this reaction. Apparently, nitroxides play more than one role in these reactions [47,48] especially the more that metal chelators were not included in our systems.

Nitroxides inhibited the oxidation of DHR123 by SIN-1, a generator of peroxynitrite, indicating that they may attenuate reactions induced by reactive nitrogen species. The effect of nitroxides might be due to the inhibition of peroxynitrite formation from its precursors (superoxide and nitric oxide) by decomposing superoxide, but this mechanism is improbable given the very high reaction rate constant between superoxide and nitric oxide [51]. Rather, this inhibition may be due to their reaction with peroxynitrite, as demonstrated by us previously for TEMPO [52].

The effect of nitroxides on the hypochlorite-induced fluorescein bleaching showed different effects for TEMPO and TEMPAMINE. While TEMPO offered significant protection, TEMPAMINE even enhanced the bleaching. This effect may be due to the formation of chloramine due to the presence of the amino group; chloramines are known to be secondary oxidants formed by hypochlorite, which may prolong the action of this compound [53,54].

At the low (≤10 μM) concentrations the nitroxides did not cause any significant toxicity to the cells studied but were also able to exert effects on their selected biochemical parameters. All the nitroxides used rapidly penetrate the plasma membrane (and other membranes), and their concentration in the extracellular medium decreased in time, due to the intracellular reduction [8,55]. The higher reduction rates of nitroxides induced by TAU than by EP cells are seemingly due to the compensatory increase in the content of antioxidants in oxidatively stressed TAU cells, as demonstrated for GSH [42].

The nitroxides decreased the level of glutathione in both cell lines, which is a prooxidant effect. Nitroxides react with glutathione slowly, with the intracellular reduction of nitroxides being due mainly to intracellular ascorbate. However, glutathione may regenerate oxidized ascorbate or ascorbate radical [56]. Glutathione depletion may appear improbable when taking into account the presence of glutathione at millimolar concentrations inside the cells [57]. However, it should be taken into account that cells in the culture constitute a small volume fraction with respect to the medium. Assuming a diameter of SH-SY5Y cells of 12 μm (https://bionumbers.hms.harvard.edu/bionumber.aspx?s=n&v=0&id=108938, accessed on 1 November 2023), one obtains a cell volume of ca 900 μm^3^. Cells seeded into a well (40,000) would occupy a volume of 32 × 10^6^ m^3^ = 3.2 × 10*^−^*^2^ mm^3^ (or μL). If a nitroxide is present at a concentration of 1 μM in 100 μL of the medium, the number of its molecules in a well is about 6.02 × 10^23^ × 10*^−^*^6^ × 10*^−^*^4^ = 0.6 × 10^14^, while if glutathione is present inside the cells at a concentration of 7 mM [57], the number of its molecules in a well is about 6.02 × 10^23^ × 7 × 10*^−^*^3^ × 3.2 × 10*^−^*^2^ × 10*^−^*^6^ = 1.3 × 10^14^, and so these numbers are comparable. This simple calculation shows also that in some in vitro experiments cells are exposed to a vast excess of nitroxides when millimolar concentrations of these compounds are employed.

The disturbance of the cellular redox homeostasis by nitroxides differently affected the parameters measured in both cell lines. The basal GSH content was about 60% higher in TAU cells than in EP cells [42]. The diminution of the GSH level by the nitroxides still left the GSH level in TAU cells higher by about 30% as compared with EP cells, which, apparently, was sufficient enough to prevent an increase in the ROS level that occurred in EP cells, presumably due to lower scavenging by the lowered level of GSH. ROS scavenging by nitroxides can account for the lowering of ROS level in TAU cells but could not compensate for the lowered scavenging by GSH in EP cells.

The basic level of lipid peroxidation and mitochondrial-membrane potential was lower in TAU cells than in EP cells. The lowered lipid peroxidation in TAU cells is seemingly due to the enhanced antioxidant defense, due to adaptation to permanent oxidative stress. The depletion of intracellular antioxidants by nitroxides seems to be responsible for the increase in lipid peroxidation in TAU cells subject to permanent oxidative stress; this effect might be not so important for the not-oxidatively-stressed EP cells.

All the nitroxides used increased the mitochondrial-inner-membrane potential in TAU cells. This effect is somewhat surprising since it could be expected that TEMPAMINE, which is partly positively charged at a close to neutral pH, would be preferentially accumulated in the mitochondria, and perhaps act as a protonophore, decreasing the mitochondrial potential. Such an effect was not observed.

The lower value of the mitochondrial-membrane potential in TAU cells than in EP cells may also be an adaptation to oxidative stress, as the rate of superoxide release decreases with decreasing mitochondrial-membrane potential [58]. Scavenging of ROS by nitroxides in TAU cells may allow for increasing membrane potential without the danger of elevating the level of ROS in TAU cells.

The present results demonstrate that nitroxides, even at low micromolar concentrations, may exert significant effects in both pure chemical systems and on cells in in vitro culture. Nitroxides at low concentrations showed no significant cytotoxicity, and the cellular effects of the three nitroxides studied were similar. However, their substituents may cause differences in their behavior in some situations, like in the case of hypochlorite action.

Cellular effects of low concentrations of nitroxides may have relevance for the application of nitroxides in the form of redox nanoparticles [59,60]. These nanoparticles, containing nitroxides as the redox-active residues, can penetrate the cells and resist in the cells for a while, but their penetration efficiency and thus intracellular concentrations arelimited so this situation may not be much different from the conditions used in this study when cells were exposed to low but sustained nitroxide concentrations. As nitroxide-based redox nanoparticles seem to be a promising therapeutic tool [60,61,62], the cellular effects of low nitroxide concentrations need a comprehensive understanding.

## 4. Materials and Methods

### 4.1. Reagents, Equipment and Cells

2,2′-Azino-bis (3-ethylobenzthiazoline-6-sulfonic acid) (ABTS; CAS no. 504-14-6; cat. no. 10102946001; purity ≥ 99%) was provided by Roche (Warsaw, Poland). 2,2-Diphenyl-1-picrylhydrazyl (DPPH; CAS no. 1898-66-4; cat. no. HY-112053; purity ≥ 99.13%), as well as iron(III) chloride (CAS no. 7705-08-0; cat. no. 451649; purity ≥ 99.99%), were obtained from MedChemExpress (Monmouth Junction, NJ, USA). 2,4,6-Tris(2-pyridyl)-s-triazine (TPTZ; CAS no. 3682-35-7; cat. no. T1253; purity ≥ 98%) and Mohr’s salt [ammonium iron(II) sulfate hexahydrate] (CAS no. 7783-85-9; cat. no. 203505; purity of 99.997%) were from Merck (Poznań, Poland). 2,2-Azobis(2-amidinopropane) dihydrochloride (AAPH; CAS no. 2997-92-4) was purchased from Polysciences (Warrington, PA, USA). A stock solution of AAPH was freshly prepared in PBS before each experiment. Dihydrorhodamine 123 (CAS no. 09244-58-8), a cell-permeable fluorogenic probe that is useful for the detection of reactive oxygen species (ROS) (DHR123; cat. no. D23806) was obtained from Thermo Fisher Scientific (Waltham, MA, USA). Ethanol (CAS no. 64-17-5; cat. no. 396480111, purity ≥ 99.8%), methanol (CAS no. 67-56-1; cat. no. 6219900110), copper (II) sulfate pentahydrate (CAS no. 7758-99-8; cat. no. 658310422, purity ≥ 98%), and sodium acetate anhydrous (CAS no. 127-09-3; cat. no. BN60/6191, purity ≥ 99%) were from Avantor Performance Materials Poland (Gliwice, Poland).

Acetic acid (CAS no. 64-19-7; cat. no. 425687339, purity 80%), hydrochloric acid (CAS no. 7647-01-0; cat. no. 115752837, 35–38%), Tris-HCl (CAS no. 77-86-1; cat. no. 118534707, purity ≥ 99%), and Folin–Ciocalteu’s reagent (cat. no. 116943507) were provided by Chempur (Piekary Śląskie, Poland).

G418 disulfate salt (CAS no. 108321-42-2; cat. no. A1720), 2-propanol (CAS no. 67-63-0; cat. no. I9516), Thiazolyl Blue Tetrazolium Bromide (MTT; CAS no. 298-93-1; cat. no. M2128), dihydroethidium (DHE; CAS no. 104821-25-2; cat. no. 37291), dimethyl sulfoxide (DMSO; CAS no. 67-68-5; cat. no. D2438), Trypan Blue solution (0.4%, liquid, sterile-filtered, suitable for cell culture; CAS no. 72-57-1; cat. no. T8154), TEMPO (CAS no. 2564-83-2; cat. no. 214000), TEMPOL (4-hydroxy TEMPO; CAS no. 2226-96-2; cat. no. 176141), TEMPAMINE (4-amino-TEMPO; CAS no. 14691-88-4; cat. no. 163945), N-ethylmaleimide (NEM; CAS no.128-53-0; cat. no. E3876), neocuproine (CAS no. 484-11-7, cat. no. N1501), trichloroacetic acid (TCA; CAS no. 76-03-9; cat. no. T4885), diethylenetriaminepentaacetic acid (DTPA; CAS no. 67-43-6; cat. no. D1133), L-ascorbic acid (CAS no. 50-81-7; cat. no. A0278), and *ortho*-phtaldialdehyde (OPA; CAS no. 643-79-8; cat. no. P1378) were provided by Merck (Poznan, Poland).

JC-1 Mitochondrial Membrane Potential Assay Kit (cat. no. ab113850) was obtained from Abcam (Cambridge, UK). Dulbecco’s modified Eagle’s medium/Nutrient Mixture F-12 without phenol red (cat. no. 21041025), Dulbecco’s phosphate-buffered saline (DPBS; cat. no. 14040-117), and Lipid Peroxidation Sensor [(4,4-difluoro-5-(4-phenyl-1,3-butadienyl)-4-bora-3a,4a-diaza-s-indacene-3-un-decanoic,] (C11-BODIPY^®^581/591; CAS no. 217075-36-0, cat. no. D3861) were purchased from Thermo Fisher Scientific (Waltham, MA, USA).

Fetal bovine serum (cat. no. S1813), penicillin–streptomycin solution (cat. no. L0022), Trypsin–EDTA solution (10×) (cat. no. X0930), and phosphate-buffered saline without Ca^2+^ and Mg^2+^ (cat. no. P0750) were obtained from Biowest (Nuaillé, France). Distilled water was purified using a Milli-Q system (Millipore, Bedford, MA, USA).

Cell culture 75 cm^2^ flasks (cat. no. 658175), transparent 96-well Advanced TCTM culture plates (cat. no 655980), black 96-well flat bottom µClear^®^ Advanced TCTM plates (cat. no. 655986), transparent 96-well (cat. no. 655101), black 96-well flat bottom plate (cat. no. 655209), and 24-well cell culture transparent plates (cat. no. 662160) were obtained from Greiner Bio-One (Kremsmünster, Austria). Other sterile cell culture materials were provided by Nerbe (Winsen, Germany), ThermoFisher Scientific (Waltham, MA, USA) or Greiner Bio-One (Kremsmünster, Austria).

The human neuroblastoma cell line derived from a metastatic bone tumor of a 4-year-old cancer patient [SH-SY5Y (CRL2266, American Cell Culture Collection) stably transfected with the longest human 4-repeated tau isoform subcloned into the pcDNA3.2/V5/DEST vector (hTau40; TAU cells)], and cells transfected with the empty vector (pcDNA3.2; EP cells) that were used as control were kindly provided by Margaret Fahnestock (McMaster University, Hamilton, ON, Canada). Both transfection groups were cultured under selective pressure with 300 µg/mL of G418.

Stock solution of TEMPO was freshly prepared in DMSO and diluted in the cell culture medium. TEMPOl, TEMPAMINE, and MTT were dissolved in PBS, filtered through a 0.22 μm filter before each experiment, and diluted in a cell medium. Distilled water was purified using a Milli-Q system (Millipore, Bedford, MA, USA).

Fluorometric and absorptiometric measurements were conducted in a Spark multimode microplate reader (Tecan Group Ltd., Männedorf, Switzerland). Transmission-light microscope observations were conducted in an inverted Olympus CKX53 microscope (OLYMPUS, Tokyo, Japan).

### 4.2. Evaluation of the Antioxidant Activity of Nitroxides

#### 4.2.1. ABTS^•^ Decolorization Assay

A modified [63] ABTS^•^ decolorization assay of Re et al. [64] was employed. Briefly, various volumes of nitroxide solutions were added to wells of a 96-well plate, containing 200 μL of ABTS^•^ solution diluted with PBS to provide absorbance of 1.0 at 734 nm in a plate reader. The decrease in absorbance was read after 30 min at ambient temperature.

#### 4.2.2. DPPH^•^ Decolorization Assay

The 2,2-diphenyl-1-picrylhydrazyl (DPPH^•^) decolorization assay was completed as described previously [65]. Briefly, aliquots (200 µL) of 0.3 mM DPPH^•^ solution in methanol were added with various volumes of nitroxide solutions or Trolox and incubated for 30 min in the dark at ambient temperature. The decrease in absorbance at 517 nm was then measured.

#### 4.2.3. FRAP Assay

The assay was performed according to a modified procedure of Benzie and Strain [66]. In brief, various volumes of nitroxide solutions were added to wells of a 96-well plate containing 200 μL of the working solution freshly prepared by mixing ten volumes of 0.3 M acetate buffer, pH 3.6; one volume of 10 mM 2,4,6-tris(2-pyridyl)-s-triazine (TPTZ) in 40 mM HCl; and one volume of 20 mM FeCl_3_. After 30 min incubation at ambient temperature, absorbance was measured at 593 nm against a reagent blank.

#### 4.2.4. CUPRAC Assay

A modification of the procedure of Özyürek et al. [67] was used for the CUPRAC assay. Briefly, 50 μL of 50 mM Tris-HCl buffer, pH 7.0, were mixed with 50 μL of 10 mM CuSO_4_, 50 μL of 7.5 mM neocuproine solution in ethanol, and 50 μL of PBS containing increasing amounts of a sample. After 60 min incubation at ambient temperature, absorbance was measured at 450 nm against a reagent blank.

#### 4.2.5. Calculation of Antioxidant Activity

In all cases, Trolox was used as a standard antioxidant, and antioxidant activity was expressed in relation to Trolox, in moles of Trolox equivalents (TE)/mol). Moreover, in all cases where the consumption of indicator radicals or formation of Fe^2+^ could be quantified from absorbance measurements, the number of moles of the indicator radicals/ions per mole of a nitroxide was calculated.

### 4.3. “Reversed FRAP” Assay

A “reversed FRAP” assay using Fe^2+^ instead of Fe^3+^ was used to detect the ability of nitroxides to oxidize Fe^2+^ ions. The reaction medium contained 80 mM Tris-HCl buffer, pH 7.0, Mohr’s salt (100 μM in Fe^2+^), and 40 or 75 μM nitroxides (final concentrations). After 20 min incubation at ambient temperature, TPTZ was added to a final concentration of 250 μM and absorbance was measured at the wavelength of 593 nm after 5 min.

### 4.4. Cell Culture

hTau40 and pcDNA cells were grown in DMEM/F12 medium without Phenol Red enriched with 1% *v*/*v* penicillin and streptomycin solution, 300 μg/mL G418, and 10% heat-inactivated foetal bovine serum (FBS). Cells were cultured at 37 °C, under 5% carbon dioxide and 95 °C humidity, and trypsinized after reaching 90% confluence. Cell morphology was examined under an inverted microscope with phase contrast Zeiss Primo Vert (Oberkochen, Germany). The viability of the cells was estimated using the Trypan Blue exclusion test. Cells were counted in a Thoma hemocytometer (Superior Marienfeld, Lauda-Königshofen, Germany).

### 4.5. Assessment of Nitroxide Cytotoxicity

TAU and EP cells were seeded onto 96-well, clear, Advanced TM plates at a density of 4 × 10^4^ cells/well, in a 100 µL culture medium and allowed to attach at 37 °C for 24 h. Subsequently, the cells were treated with TEMPO, TEMPOL, and TEMPAMINE at concentrations of 1, 2.5, 5, or 10 µM. Working solutions of the studied factors were prepared in the culture medium. The DMSO concentration, if used, was adjusted to 0.2% in all samples, including controls. This concentration had no significant effect on the treated cell lines. After 24 h exposure time, the medium was removed and replaced with 100 µL of 0.5 mg/mL MTT solution in PBS and incubated for 2 h at 37 °C. After incubation, 100 µL/well of acidic isopropanol (250:1 isopropanol:HCl) was added, and the contents of the well were thoroughly mixed. The plate was shaken (700 rpm) at room temperature for 20 min. Absorbance was measured at 570 nm.

### 4.6. Reduction of Nitroxides by Cells

TAU and EP cells were seeded into 96-well, clear, Advanced TM plate at a density of 4 × 10^4^ cells/well, in a 100 µL culture medium and allowed to attach at 37 °C for 24 h. Then, the medium was removed and replaced by 100 µL of a new medium containing 10 µM nitroxide. Immediately, and after 5, 10, 20, 40, and 60 min incubation at 37 °C with intermittent shaking, the medium was aspirated from subsequent wells, and the EPR signal of the nitroxides was measured in the aspirated medium.

Electron paramagnetic resonance (EPR) measurements were performed in a Bruker multifrequency and multiresonance FT-EPR ELEXSYS E580 spectrometer (Bruker Analytische Messtechnik, Rheinstetten, Germany), operating at the X-band (9.837530 GHz). The following settings were used: central field, 3505.6 G; modulation amplitude, 1 G; modulation frequency, 100 kHz; microwave power, 94.64 mW; power attenuation, 10 dB; scan range, 80 G; conversion time, 25 ms; and sweep time, 25.6 s.

### 4.7. The Level of Reactive Oxygen Species

The level of ROS in neuroblastoma cells after being treated with different concentrations of nitroxides was assayed with dihydroethidium (DHE). The cells were seeded in 96-well, flat, clear-bottom black plate at an amount of 4 × 10^4^/well and allowed to attach at 37 °C for 24 h. Subsequently, the cells were treated with the nitroxides for 24 h. After, the incubation medium was removed and replaced by 10 µM DHE in PBS (100 µL/well). A stock solution of the probe was prepared in DMSO, and the working solution was prepared by dilution with PBS. Fluorescence was measured at 475/579 nm at 37 °C every minute for 2 h. The sum of fluorescence values was taken as a measure of ROS production.

### 4.8. Estimation of the Content of Reduced Glutathione

The content of reduced glutathione was estimated with *ortho*-phtalaldehyde (OPA), according to Senft et al. [68]. The cells were seeded in a 96-well clear plate at an amount of 4 × 10^4^/well and allowed to attach at 37℃ for 24 h. After 24 h treatment with the nitroxides, the medium was removed, cells were washed with PBS (150 µL per well) and then 60 µL/well of the cold Redox Quenching Buffer (RQB) containing 20 mM HCl, 5% trichloroacetic acid (TCA), 5 mM diethylenetriaminepentaacetic acid (DTPA), and 10 mM L-ascorbic acid were added. The plate was shaken for 5 min and centrifuged at 4000 rpm for 5 min. Next, the cell lysates were transferred into two 96-well black-bottom plates (+NEM and −NEM) in an amount of 25 µL/well. Within the plate, +NEM 4 µL/well of freshly prepared 7.5 mM NEM in cold RQB buffer was added. Then, 40 µL/well 1 M phosphate buffer (pH 7) was added to both plates, and the plates were shaken at 700 rpm for 5 min. Subsequently, 160 µL/well of cold 0.1 M phosphate buffer (pH 6.8) and 25 µL/well of freshly prepared 0.5% OPA in methanol were added to both plates. The plates were incubated for 30 min at room temperature under constant stirring. Fluorescence was measured at 355/430 nm. Protein content in cell lysates was determined according to Lowry et al. [69]. The concentration of reduced glutathione was determined by subtracting the fluorescence of the (−NEM) plate from the fluorescence (+NEM) plate and dividing by the protein content.

### 4.9. Lipid Peroxidation Assay with BODIPY™ 581/591 C11

The cells were seeded on a 96-well flat clear-bottom black plate at a density of 4 × 10^4^ cells/well and allowed to attach for 24 h at 37 °C. Subsequently, cells were treated for 24 h with the nitroxides. Next, the medium was removed, the fluorescent lipid peroxidation probe C-11 BODIPY in the cell culture medium was added to the final concentration of 1 µM, and the cells were incubated for 30 min at 37 °C. From the fluorescence emission spectrum at the excitation wavelength of 460 nm, two wavelengths from the emission maximum were selected. Fluorescence was measured at 460/523 nm and 460/596 nm. The results are presented as the ratio of fluorescence intensity at 523 nm to 596 nm emission wavelengths.

### 4.10. Evaluation of Changes of the Mitochondrial-Membrane Potential (∆Ψ_m_)

Changes in the mitochondrial-membrane potential (∆Ψ_m_) were estimated with a Mitochondrial Membrane Potential Asay Kit employing 5,5*′*,6,6*′*-tetrachloro-1,10,3,30-tetraethylimidacarbocyanine iodide (JC-1). Briefly, the cells were treated with nitroxides as above, 100 µL of the medium containing 10 µM JC-1 was added to wells, and the plate was incubated at 37 °C for 30 min. Subsequently, the medium with JC-1 was gently removed, and 100 µL of buffer included in the kit was added per well. Fluorescence was measured at 540/570 nm (red fluorescence) and 485/535 nm (green fluorescence). The results were presented as the green-to-red fluorescence intensity ratio.

### 4.11. Statistical Analysis

The results are presented as means ± SD from three independent experiments. To estimate the statistical significance of differences, ANOVA was used except for simple comparisons between two groups where Student’s *t*-test was employed. *p* < 0.05 was considered statistically significant. Statistical analysis of the data was performed using the STATISTICA software package (version 13.1, Statsoft Inc. 2016, Tulsa, OK, USA).

## Figures and Tables

**Table 1:** Antioxidant activity of nitroxides in various antioxidant assays. Ascorbate was used for comparison as a standard antioxidant.

Method	Ascorbate	TEMPO	TEMPOL	TEMPAMINE
Antioxidant activity [TE/mol]				
ABTS^•^ decolorization	0.98 ± 0.06	0.20 ± 0.02	0.03 ± 0.01	0.03 ± 0.002
DPPH^•^ decolorization	0.70 ± 0.05	0.59 ± 0.06	0.53 ± 0.05	0.48 ± 0.06
FRAP	1.16 ± 0.08	0.40 ± 0.03	0.50 ± 0.08	0.30 ± 0.06
CUPRAC	0.86 ± 0.11	0.70 ± 0.27	0.44 ± 0.08	0.57 ± 0.09
Number of moles of a radical/ion reduced by mole of a nitroxide				
ABTS^•^	1.625 ± 0.031	0.291 ± 0.082	0.045 ± 0.006	0.044 ± 0.002
DPPH^•^	1.42 ± 0.07	1.20 ± 0.11	1.08 ± 0.09	0.88 ± 0.07
Fe^3+^	2.16 ± 0.07	0.85 ± 0.07	1.06 ± 0.17	0.64 ± 0.13

**Table 2:** Oxidation of Fe^2+^ by nitroxides in the reversed FRAP assay.

Nitroxide Concentration [μM]	TEMPO	TEMPOL	TEMPAMINE
Fe^2+^ oxidized [μM]			
8	18.4 ± 0.1	17.7 ± 1.2	14.2 ± 1.1 **
15	25.5 ± 0.9	28.1 ± 0.5 *	33.3 ± 0.8 ***

* *p* < 0.05, ** *p* < 0.05, *** *p* < 0.001 vs. TEMPO.

**Table 3:** Rate of loss of EPR signal of nitroxides (10 μM) incubated (37 °C) with EP and TAU cells (μM min^−1^).

Cells/Nitroxide	TEMPO	TEMPOL	TEMPAMINE
EP	0.057 ± 0.005	0.049 ± 0.009	0.054 ± 0.006
TAU	0.075 ± 0.005 *	0.068 ± 0.007 *	0.071 ± 0.008 *

* *p* < 0.05 (TAU vs. EP cells; Student’s “*t*” test).

## Data Availability

Data are available from the corresponding author upon reasonable request.
